# Assessment of Traffic-Related Air Pollution: Case Study of Pregnant Women in South Texas

**DOI:** 10.3390/ijerph16132433

**Published:** 2019-07-09

**Authors:** Mohammad Hashem Askariyeh, Suriya Vallamsundar, Josias Zietsman, Tara Ramani

**Affiliations:** 1 Environment and Air Quality Division, Texas A&M Transportation Institute, 1111 RELLIS Parkway, Suite 3401, Bryan, TX 77807, USA; 2 Zachry Department of Civil and Environmental Engineering, Texas A&M University, 201 Dwight Look Engineering Building, College Station, TX 77843, USA; 3 Center for Advancing Research in Transportation Emissions, Energy and Health, Texas A&M Transportation Institute, 12700 Park Central Dr, Suite 1000, Dallas TX 75251, USA; 4 Center for Advancing Research in Transportation Emissions, Energy and Health, Texas A&M Transportation Institute, 1111 RELLIS Parkway, Suite 3401, Bryan, TX 77807, USA

**Keywords:** dynamic exposure, vehicle emissions, air pollution, pregnant women, GPS, dispersion method, particulate matter PM_2.5_

## Abstract

Population groups vulnerable to adverse effects of traffic-related air pollution correspond to children, pregnant women and elderly. Despite these effects, literature is limited in terms of studies focusing on these groups and a reason often cited is the limited information on their mobility important for exposure assessment. The current study presents a method for assessing individual-level exposure to traffic-related air pollution by integrating mobility patterns tracked by global positioning system (GPS) devices with dynamics of air pollutant concentrations. The study is based on a pool of 17 pregnant women residing in Hidalgo County, Texas. The traffic-related particulate matter with diameter of less than 2.5 micrometer (PM_2.5_) emissions and air pollutant concentrations are predicted using MOVES and AERMOD models, respectively. The daily average traffic-related PM_2.5_ concentration was found to be 0.32 µg/m^3^, with the highest concentration observed in transit (0.56 µg/m^3^), followed by indoors (0.29 µg/m^3^), and outdoor (0.26 µg/m^3^) microenvironment. The obtained exposure levels exhibited considerable variation between time periods, with higher levels during peak commuting periods, close to the US–Mexico border region and lower levels observed during midday periods. The study also assessed if there is any difference between traffic-related dynamic exposure, based on time-varying mobility patterns, and static exposure, based solely on residential locations, and found a difference of 9%, which could be attributed to the participants’ activity patterns being focused mostly indoors.

## 1. Introduction

Recent evidence has shown traffic emissions to be a significant source of air pollution [1], with several studies showing a strong association between elevated emissions levels and near-roadway areas [2,3,4,5]. Accordingly, epidemiology studies have documented adverse respiratory and cardiovascular effects [6,7,8] for populations living in close proximity to major roadways. Studies have shown specific adverse health effects of being exposed to Particulate Matter (PM), including heart diseases [9,10], cancer risk [11] and adverse birth outcomes [12]. Children, pregnant women, elderly and people with existing health issues are some of the vulnerable population groups to adverse traffic-related air pollution. Studies have shown an association between traffic-related air pollution and reduced fetal growth, preterm birth and post-term low birth weight and susceptibility to asthma [13,14].

Exposure to air pollution depends on (1) the location and time of exposure and (2) the emission concentrations in different microenvironments. Most epidemiological studies estimate exposure levels based on data from ambient monitors and census information [15,16,17,18]. Due to the spatial movement of individuals, fixed ambient monitoring data might not accurately capture the exposure levels [19,20]. Ambient monitors give an overall estimate of pollutant levels and do not provide a source-specific or time-specific contribution, while studies have shown significant temporal and spatial variation in exposure levels [21,22]. Another drawback in traditional exposure assessment is the use of census data that assumes people are at a fixed residential location, which differs from reality because people move for work, school or recreational purposes, thereby leading to different exposure levels [23,24,25,26,27]. While people might spend most of their time indoors (e.g., home), they travel by different means of transportation to different microenvironments (e.g., work, school, shops, etc.) leading to different levels of exposure [28] compared to their fixed residential location. It is therefore important to better understand the movement of people and the time spent in the different microenvironment to better assess their exposure to air pollution.

Methods utilized for exposure assessment include proximity-based models, land-use regression, interpolation methods and air dispersion models. Each of these methods involves different levels of input data, resolution, assumptions and accuracy. Proximity method employs metrics such as traffic density and distance to roadway to assess the air pollution exposure. However, these metrics are developed based on a fixed residential location of the study population. Land-use regression and interpolation methods have been widely employed for exposure assessment [19,29,30]. However, these methods depend on the ambient monitoring stations as their source data in assessing the exposure levels of the study population and hence are limited by the number and spatial coverage of the monitoring stations [31,32]. Ideally, the most accurate way to measure exposure is by employing personal exposure monitoring, which is typically limited in terms of the sample size due to the higher cost of covering larger samples. Air dispersion models, on the other hand, use numerical techniques to simulate the dispersion patterns of air pollutants based on a particular source of emission, the rate of emission release, meteorology and land use. These models can track concentrations specific to emission sources and can capture highly resolved spatial and temporal variations of concentration estimates [33,34] However, assumptions must be made regarding their steady-state formulation; lack of capturing the chemical transformation of pollutants and resources may become intensive depending on the study area extent [35]. Several prior studies have evaluated exposure levels based on air dispersion models [36,37,38].

Among the different population groups, there have been limited studies focused on assessing maternal exposure to air pollution [39,40,41,42]. Exposure levels for pregnant women could vary compared to the general population because of their different activity patterns [40,43]. The National Human Activity Patterns Survey, an extensive survey conducted from the year 1992 to 1994 for 9386 people all over the US did not address pregnant women activity patterns [44]. Limited studies [40] have determined the activity patterns based on self-reported assessments. These assessments could lead to inaccurate reporting of location or activity duration [45]. Emerging technologies such as smartphones and Bluetooth devices are able to provide highly resolved spatial and temporal information about people’s locations [46,47]. Anonymized data obtained from these technologies has the potential to improve the characterization of emissions exposure.

Exposure levels modeled based on the dynamic location information of people are referred to as dynamic exposure compared to static exposure measured based on fixed residential location of people. The aim of the study is to model the dynamic exposure to traffic-related pollution for a sample population of pregnant women and compare it with static exposure. The study uses a variety of datasets such as the global positioning system (GPS) technology to continuously track participants’ location, air dispersion modeling and spatial interpolation techniques to model dynamic population exposure of traffic-related pollution for a group of pregnant women in South Texas. This study is conducted in Hidalgo County, Texas, where the prevalence of childhood asthma is found to be the highest in the state of Texas. The study is based on 17 third trimester pregnant women who are equipped with a portable GPS tracking device for three days. Specifically, this study focuses on exposure to fine PM with a diameter of less than 2.5 µm (PM_2.5_). PM_2.5_ presents a greater health threat than coarser PM because its small size allows penetration deep into the lung and studies have found a link between finer PM exposure and adverse birth outcomes and respiratory health conditions in children [48].

This paper summarizes the modeling framework used to estimate exposure to traffic-related air pollution based on air dispersion modeling and location information tracked by GPS devices. The key objective is achieved through the following goals. First, predict traffic-related air pollution emissions and concentrations at a refined roadway link level for the entire county. The US Environmental Protection Agency (EPA) regulatory models MOVES and AERMOD are employed for calculating the emission and pollutant concentrations, respectively. Second, examine dynamic exposure to traffic-related air pollution by combining predicted concentrations with dynamic location information obtained from the GPS devices. Third, evaluate the difference between dynamic and static exposure assessment methods. The paper is organized as follows. In Section 2, the framework of emission, air quality and exposure modeling chain is discussed, followed by the case study set-up in Section 3. Results are presented and discussed in Section 4, followed by a discussion of the limitations of the study in Section 5 and conclusions in Section 6.

## 2. Materials and Methods

**Figure 1:**
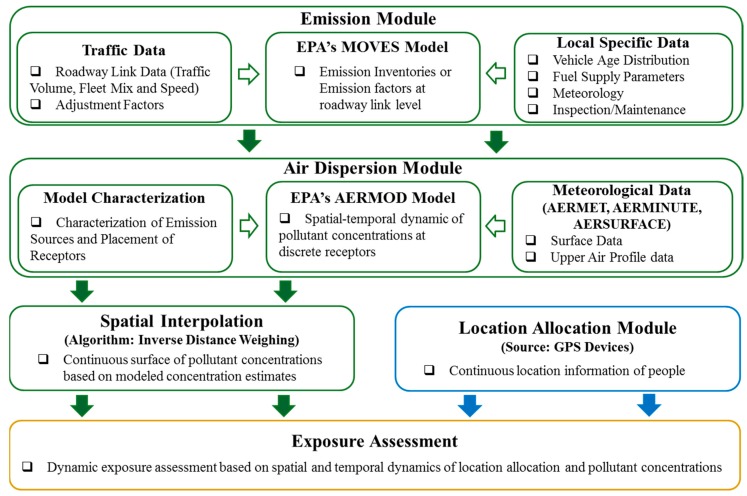
Overall modeling framework.

Figure 1 shows the flow of data in the modeling system developed for the study. Emission module calculates emission rates based on traffic and other parameters. The emission data are modeled with EPA’s microscopic MOVES emission model that utilizes site-specific traffic activity data and other local specific data corresponding to vehicle age distribution, fuel supply, inspection/maintenance parameters, etc. These emission rates are combined with meteorological and land-use data in the dispersion module. Site-specific meteorological and land-use data are obtained from closest surface data and upper air weather stations. The pollutant dispersion is modeled using the AERMOD model at discrete receptor locations. A spatial interpolation technique is applied to the discrete concentration levels to create a continuous surface of PM_2.5_ concentrations. Mobility information of participants is tracked by GPS devices for 24 hours at a 10-second resolution. Specific details about the different modeling components are discussed in this section.

### 2.1. Air Dispersion Modeling

**Figure 2:**
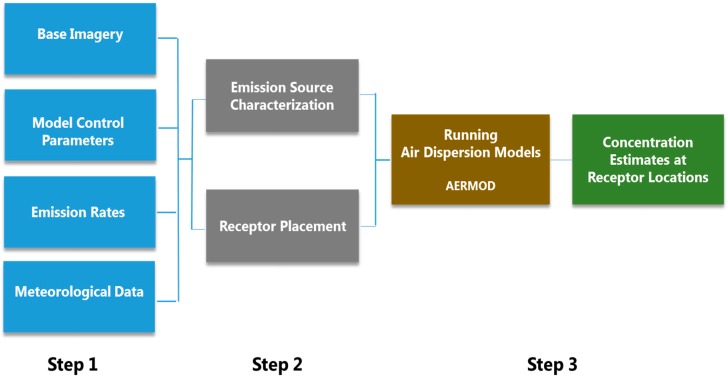
Air dispersion modeling process.

AERMOD estimates pollutant dispersion with a Gaussian-based equation, which incorporates factors that account for the rate the plume disperses in each direction, reflection from the ground and plume rise [49]. The dispersion modeling process consists of three broad steps as shown in Figure 2.

Step 1 consists of obtaining the base imagery, specifying model control parameters and securing emission, meteorological and land-use data. Base imagery shows the geographical locations corresponding to the study area and helps in geographically coding the sources and receptors. The model control parameters refer to specifying the pollutant type, pollutant properties, averaging period, etc. Three types of data are required for processing the meteorological data, namely: (1) Land-use data that represent surface characteristics, (2) surface data collected at airports by the National Weather Service (NWS) and (3) upper air sounding data collected by NWS. The land-use data is obtained from the US Geological Survey (USGS) Land Use database [50]. The raw data are processed by AERMOD preprocessors, (AERMET, AERMAP, AERMINUTE and AERSURFACE) in a format compatible for AERMOD.

Step 2 consists of characterizing the emission sources (roadway links) and placing receptors. AERMOD area source characterization is used to model the roadway links. The emission source (roadway link) characteristics are defined based on the roadway link orientation, geometry and travel activity. Pollutant concentration levels are calculated at discrete receptor locations, placed at an average adult breathing height of 1.8 m. To capture the peaking tendency of traffic-related emissions, receptors are placed at varying spacing with a higher density closer to roadways and increasing the spacing with distance from the roadways.

Step 3 is based on inputs assembled from Steps 1 and 2; AERMOD estimates pollutant concentrations at an hourly averaging period at all receptor locations.

### 2.2. Spatial Interpolation

The traffic-related pollutant concentrations estimated at discrete receptor locations are converted into a continuous surface by employing a spatial interpolation technique in a geographic information system (GIS) platform. Commonly used spatial interpolation algorithms include inverse distance weighing (IDW) [51], kriging [52], shape functions [53] and trend surfaces [54]. Furthermore, IDW is one of the most commonly used technique for exposure assessment [55,56,57]. The inverse distance weighting (IDW) technique is utilized, which estimates the value at an unknown location based on computed values at nearby locations. Closer locations (or receptors) are given more weight than those farther away, and the weight rate of decrease with distance is dependent on the power value (a power value of 2 is used in this study).

### 2.3. Location Allocation

The spatial–temporal dynamics of a participant’s location is captured using portable GPS devices. The advantages of using portable GPS devices include minimum burden for participants, high-resolution continuous tracking of location and reduction of human errors in reporting the locations [58,59]. The location information is overlaid over the continuous concentration maps generated by AERMOD to obtain dynamic exposure levels of the participants across the study area.

### 2.4. Exposure Assessment

Early researchers [60,61] first established the mathematical formulation (Equation (1)) for exposure assessment as the product of time and concentrations in different locations.

(1) E=∫C(t)dt

where *E* is the cumulative exposure (concentration × time), *C(t)* is the traffic-related pollutant concentration as a funtion of time and t is the time spent in different locations. Time-weighted average exposure is estimated by dividing *E* by *T* (total time spent in all locations) [62]. Three classifications of microenvironments are considered in this study. Incorporating these microenvironments, Equation (1) is customized into Equation (2), considering indoors, outdoors and in-vehicle [63].

(2) $$E_{i}=\sum \left(T_{i\;}C_{i\;}+T_{o\;}C_{o\;}+T_{v\;}C_{v\;}\right)$$

where *C_i_*, *C_o_* and *C_v_* are the concentration levels measured indoors, outdoors and in-vehicle, respectively; and *T_i_*, *T_o_* and *T_v_* are the corresponding time durations. Concentrations predicted by the model correspond only to the ambient concentrations, and concentrations in other microenvironments (indoors and in-vehicle) depend on factors related to the pollutant type and its penetration rate, air exchange rate inside the building and traffic conditions (idling, windows opened/closed) etc. To incorporate the changes in concentrations for each microenvironment, Equation (2) is modified into Equation (3). The adjustment factors are used to account for the difference in indoor and in-vehicle concentration compared to ambient concentration.

(3) $$E=\sum \left(C_{i\;}T_{i\;}A_{i}+\;C_{o\;}T_{o\;}+C_{v\;}T_{v\;}A_{v}\right)$$

where *A_i_* and *A_v_* are the adjustment factors for indoor and in-vehicle locations, respectively. The adjustment factors are obtained from an assessment of studies in literature that developed these factors specific to PM_2.5_, as shown in Table 1 [63]. Average values of the adjustment factor of 0.88 and 1.79 are used for indoor and in-vehicle microenvironments, respectively. Using this approach, cumulative exposure is calculated as a function of the person, time and microenvironment.

## 3. Case Study

**Figure 3:**
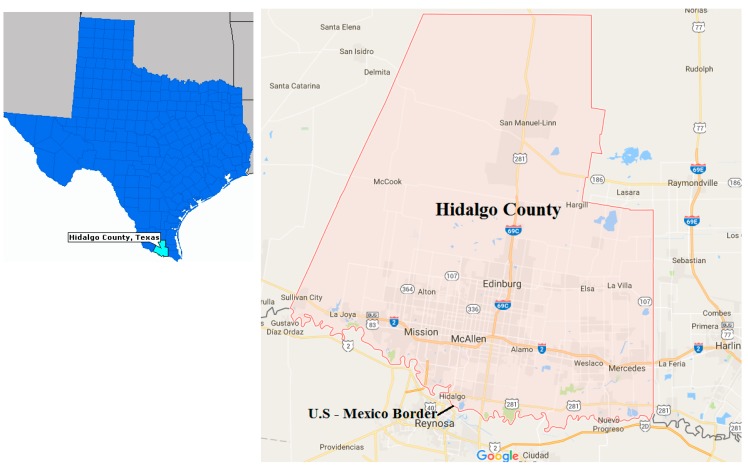
Case study location.

The case study is located in Hidalgo County, Texas (shown in Figure 3). As of the 2017 census, the county has an estimated population of 860,661, making it the eighth-most populous county in Texas. The county has the highest prevalence of childhood asthma in the State and accounts for the greatest share of people receiving food stamps [71]. The largest city in Hidalgo is McAllen, while the county seat is Edinburg.

Study participants consisted of women in their third trimester of pregnancy recruited from the Rio Grande Valley Regional OBGYN Clinics in McAllen and Edinburg, TX. A pool of 17 participants carried a portable GPS device from October 2015 to May 2016 for three non-consecutive 24-h periods. This resulted in a total of 50 sampling days (16 participated in the sampling on days and one conducted the sampling in two days).

**Figure 4:**
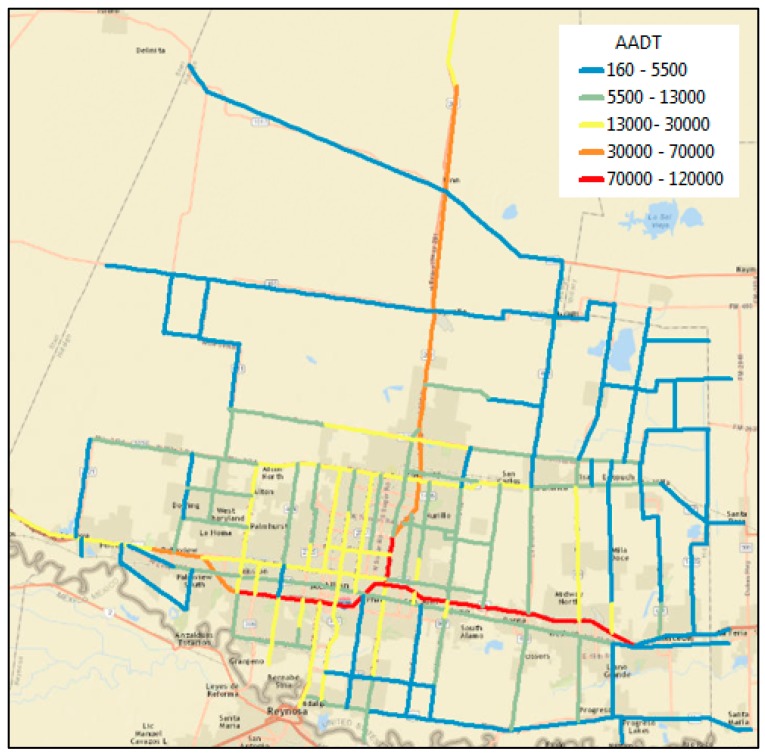
Traffic activities (annual average daily traffic (AADT)) across the study extent.

The PM_2.5_ traffic-related emissions and concentration levels are modeled for the entire Hidalgo County. The traffic activity data for all roadway links (excluding minor collectors and local roads) is obtained from traffic counters maintained by the Texas Department of Transportation TxDOT [72], as shown in Figure 4. The activity data collected included average daily traffic volumes, average vehicle trajectory and fleet composition at the roadway link level. The annual average daily traffic (AADT) is converted into hourly volumes using adjustment factors and hourly traffic percentages. These factors are developed based on regional information (related to vehicle age distribution, fuel supply and inspection/maintenance program) [73]. Traffic levels are found to be higher during morning and evening peak commuting periods and lower during overnight time periods.

The composite PM_2.5_ emission inventories for all roadway links are obtained from the latest version of EPA’s MOVES emission model. Surface data was obtained from the McAllen International Airport and the upper air data from the Brownsville Airport. The predominant wind direction is found to be from the south-east to north-west direction.

**Figure 5:**
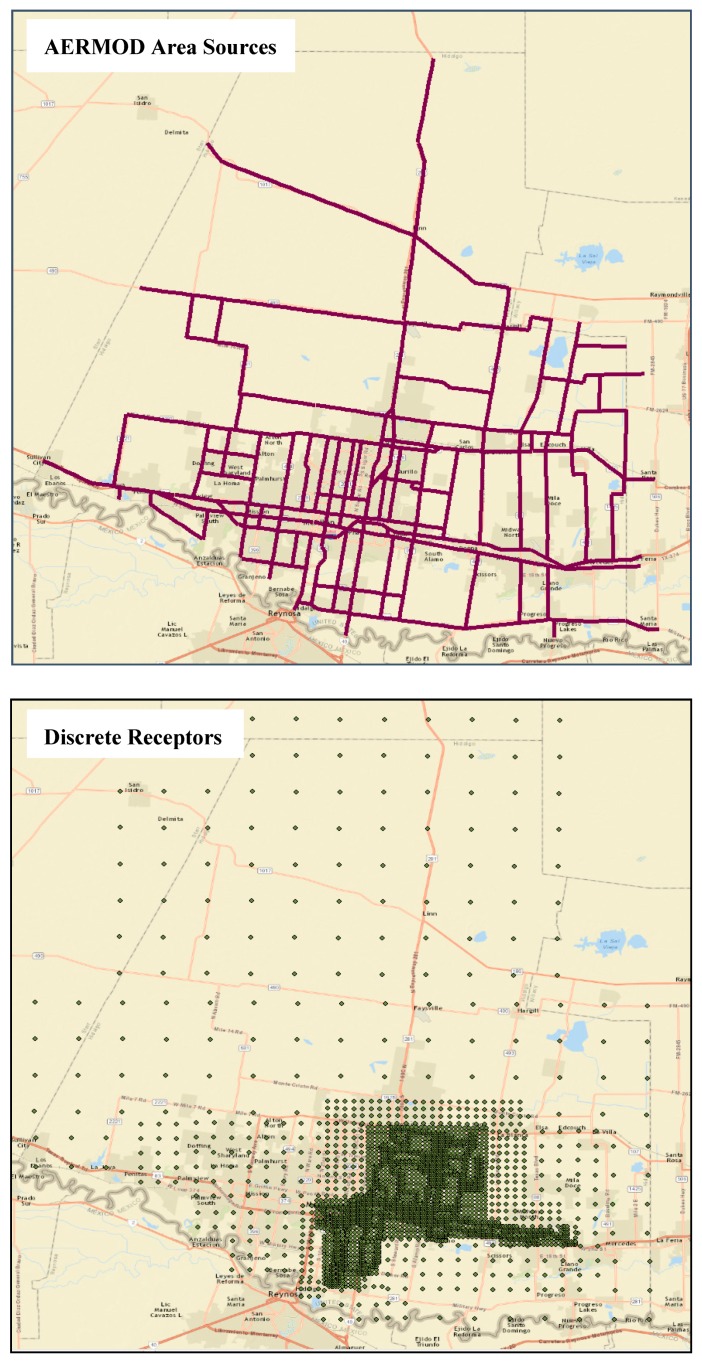
AERMOD emission source and receptor placement for case study site.

The roadway links are modeled as a series of area sources in AERMOD depending on the traffic volumes and link geometry to ensure each source has the same volume and the geometry of the links are preserved. Concentration estimates are obtained at discrete receptors placed at a height of 1.8 m and a density of 50 m near the urban core area which is increased to 500 m away resulting in a total of 3500 receptors for the study area (Figure 5). The concentration levels are estimated at every receptor location at an hourly averaging period for 50 sampling days when the participants carried the GPS devices. Continuous surfaces of PM_2.5_ concentrations are developed with IDW interpolation based on estimates at discrete receptor locations. Concentration maps generated for a total of 1200 h (50 days × 24 h) are then combined with the location information of participants to assess their dynamic exposure. The spatial and temporal coordinates contained in the GPS datasets are used to identify the location and time spent by each participant in different microenvironments. Concentration data are extracted from the concentration maps by matching the location and time contained in the GPS dataset. Exposure values are calculated according to Equation (3) for all time steps within a day and are averaged to obtain the participant’s average hourly and daily exposure.

## 4. Results

### 4.1. Exposure to Traffic-Related PM_2.5_ across Different Microenvironments

**Figure 6:**
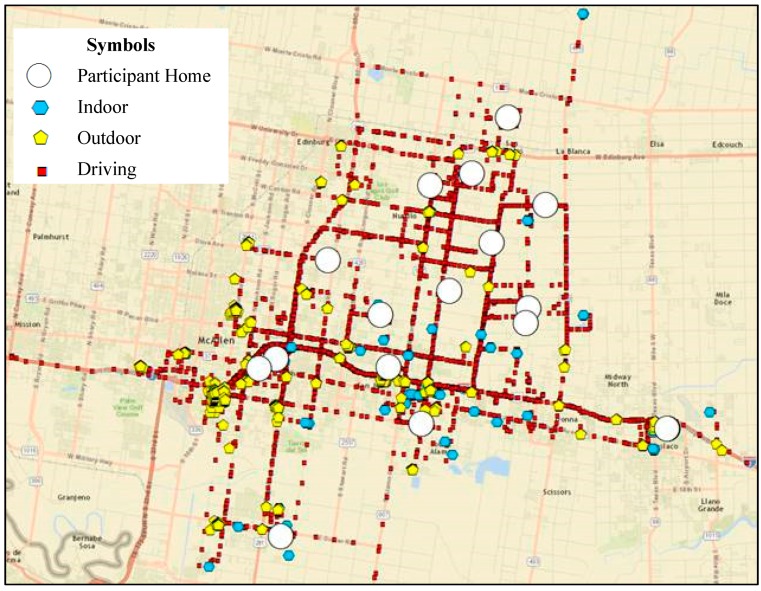
The time location trace from GPS coordinates for all 17 participants (50 sampling days).

The GPS device tracked the location of the participants, which helped for accurate microenvironment classification. The process of converting the location information is described elsewhere [74]. The case study extent and the participant’s location tracked for all sampling days are shown in Figure 6. Color points categorize the participant’s location as blue for indoor, yellow for outdoor and red for in-vehicle microenvironments. The white circles indicate participant’s residential location and have a high percentage of recorded locations indicating most of the time spent at home. In total, participants are found to spend 6.8%, 88.1% and 5.1% time outdoors, indoors and in in-vehicle microenvironments, respectively.

**Figure 7:**
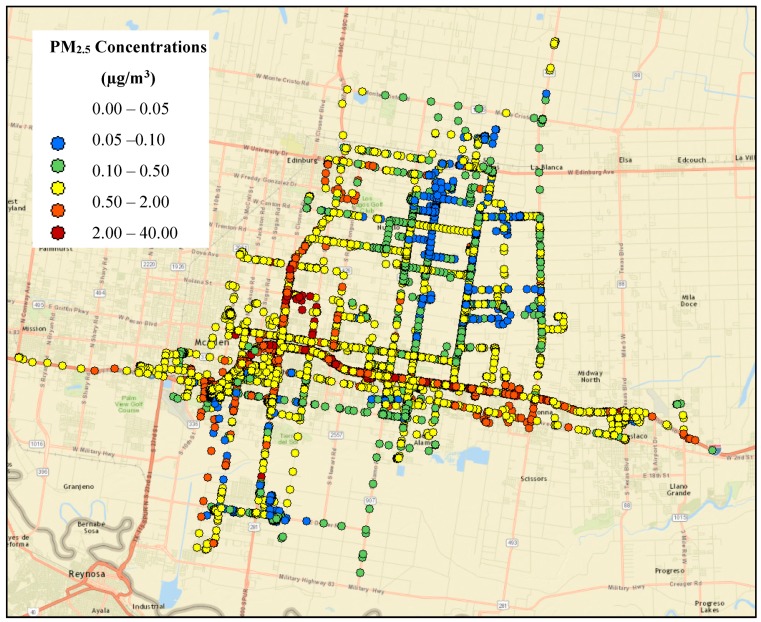
The traffic-related PM_2.5_ mass concentration (µg/m^3^) modeled by AERMOD as a function of time and GPS coordinates for all 17 participants.

The traffic-related PM_2.5_ concentration map is shown in Figure 7. Matching the concentration levels with the participant’s location indicates higher levels of PM_2.5_ exposure along the driving location trace. This implies that participants experienced relatively higher exposure when they are traveling compared to the other microenvironments. This finding highlights the importance of incorporating the participant’s location information in exposure assessment to identify if any short-term exposure (such as commuting) contributed significantly to overall exposure levels.

Statistics of the modeled exposure to traffic-related PM_2.5_ over the sampling period are shown in Table 2. Average daily in-vehicle PM_2.5_ concentration ranged between 0.02 and 1.04 µg/m^3^, with a mean value of 0.32 µg/m^3^. The average concentrations obtained in this study are relatively low compared to other studies in the literature [63]. The predicted traffic-related PM_2.5_ concentrations are attributed to the low levels of PM_2.5_ emissions from the transportation sector in comparison to other sources in the case study region. According to the EPA’s emission-based source sector classification for Hidalgo County, the region is affected predominantly by dust and agricultural sources, while mobile source emissions account for 7%.

The exposure levels vary depending on the microenvironment visited and amount of time spent in the microenvironment. The exposure intensity (or mass-to-time ratio) is obtained to determine the relative contribution of each microenvironment to the total exposure. The ratio is obtained by dividing the mass concentration in each microenvironment by the time spent in the microenvironment. The ratios are estimated to be 0.91, 1.45 and 1.96 for indoor, outdoor and in-vehicle microenvironments, respectively. The in-vehicle mass-to-time ratio is found to be doubled compared to the indoor microenvironment. This could be attributed to the close proximity to the emission source (roadway links) and the shielding offered by the buildings.

**Figure 8:**
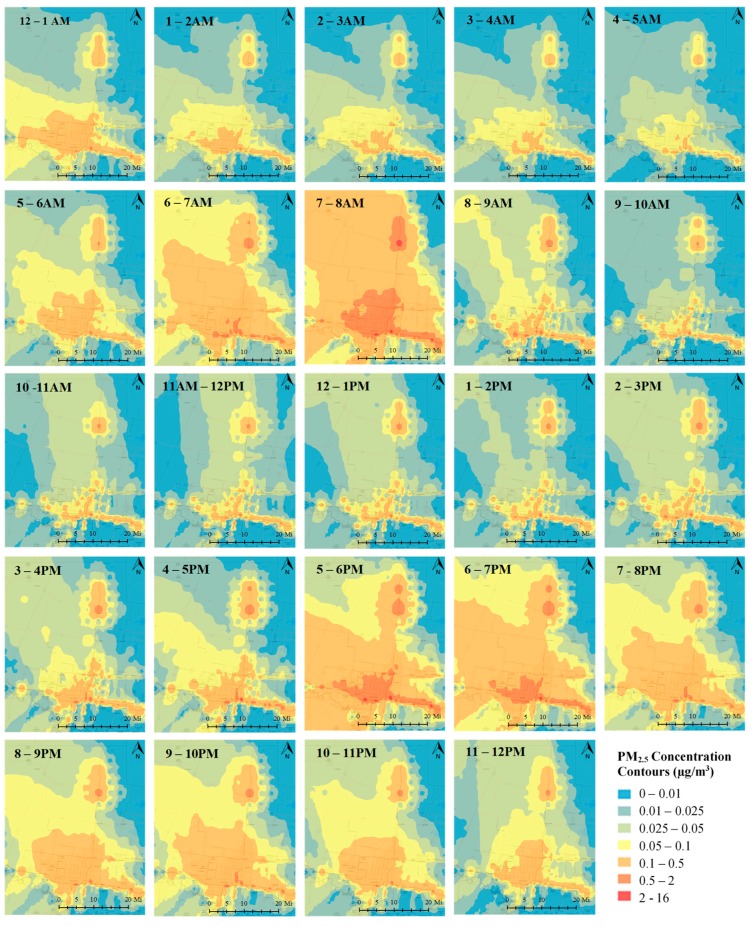
Spatial–temporal distribution of traffic-related PM_2.5_ for a sampling day on December 15, 2015.

The temporal and spatial distribution of traffic-related exposure for one sampling day (December 15, 2015) is shown in Figure 8. Higher concentration levels are observed near roadway links carrying higher traffic volumes. These links correspond to Interstate 2, one of the major east-west routes that traces the US–Mexico border and 69C which connects to the Mexican Federal Highway 97. The traffic and meteorological conditions have a dominating effect on the distribution of concentration levels. Key meteorological parameters governing the dispersion correspond to atmospheric stability and wind speed and direction. The higher PM_2.5_ concentrations predicted during peak morning (5:00–8:00 a.m.) and evening (5:00–7:00 p.m.) traffic hours are associated with higher traffic activities during these time periods. Typically, higher concentrations are found to coincide with the direction of the prevailing or dominant wind. In terms of wind speed, higher speed results in higher dilution, thereby reducing the concentration estimates. Atmospheric stability characterized by a continuous measure of Monin-Obukhov length in AERMOD can be broadly classified into unstable, stable and neutral conditions [33]. Unstable conditions, occurring during early afternoon periods, decrease the concentration levels by increasing the atmospheric mixing effect. Alternatively, due to reduced atmospheric dispersion, stable conditions common during nighttime periods increase the concentration levels and neutral conditions are between stable and unstable conditions. Specific to the case study, concentration levels during early morning periods, despite lower traffic conditions, are found to be higher which can be attributed to the stable atmospheric conditions. Lower concentration levels are observed during midday periods (10:00 a.m.–4:00 p.m.) due to a combination of low traffic volumes and unstable atmospheric conditions.

### 4.2. Static and Dynamic Exposure

**Figure 9:**
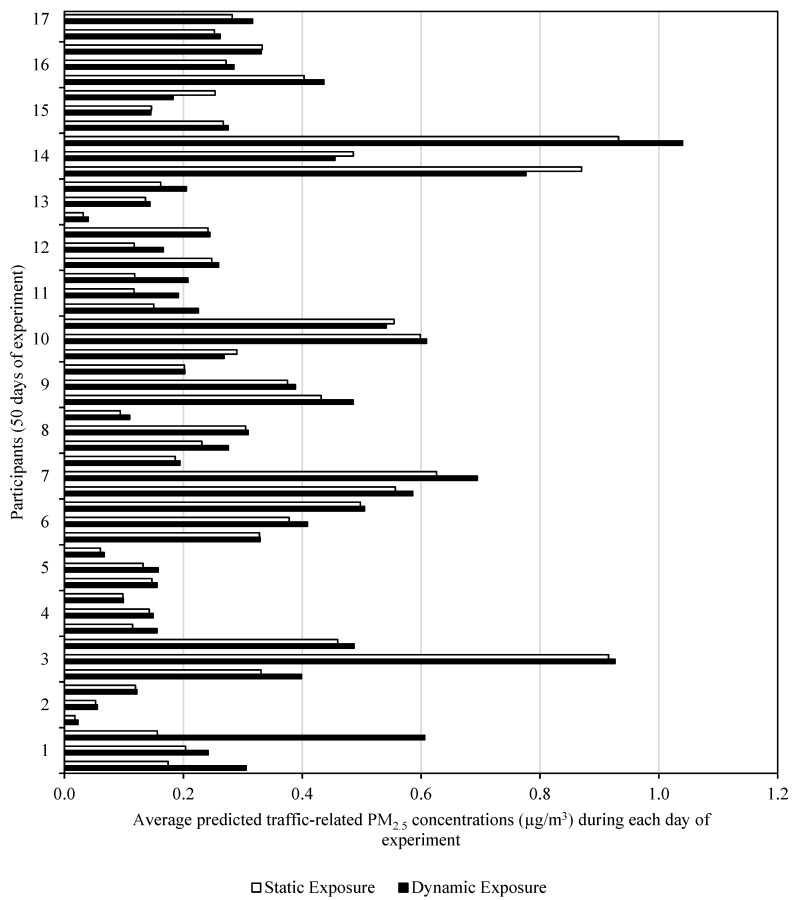
Static and dynamic exposure measures of traffic-related PM_2.5_ concentrations (µg/m^3^).

The dynamic exposure levels are calculated according to Equation (3), which reflects the location of the participant and the amount of time spent at different locations, and modeled concentrations at the corresponding location. To evaluate the impact of incorporating dynamic location information of participants in exposure assessment, an additional analysis is performed based on fixed residential locations. The static approach adopted by a number of exposure studies assumes people to be at fixed residential locations [15,16,17,18,63]. The dynamic approach, on the other hand, takes into account the dynamic location information of people for calculating their exposure levels. The distribution of static and dynamic exposure is shown in Figure 9. The average 24-h static and dynamic exposure to traffic-related PM_2.5_ over the sampling period is found to be 0.29 µg/m^3^ (95% CI = 0.23–0.35 µg/m^3^) and 0.32 µg/m^3^ (95% CI = 0.26–0.38 µg/m^3^), respectively. The results indicate that, for the entire sample, mean dynamic exposure is 9% higher than the mean static exposure. However, the difference between predicted static and dynamic exposure to traffic-related PM_2.5_ is not statistically significant (*p*-value > 0.05). The reason for the modest difference between static and dynamic exposure assessments can be explained by the activity patterns of the participants considered for the current study. These participants, being in their third trimester of pregnancy, were predominantly at their residential locations as indicated by their GPS coordinates.

A closer look at Figure 9 reveals some details about the importance of dynamic exposure predictions in exposure assessments in urban areas. The predicted dynamic exposure to traffic-related air pollution is generally expected to be greater than the static exposure because people get out of their homes for work/school/recreational purposes and get closer to a roadway transport mode. However, static exposure can be greater than dynamic exposure in some cases, like what can be seen for participant 14 (Figure 9). For this particular participant, the predicted static exposure to traffic-related PM_2.5_ is greater than most of the other participants for two out of three sampling days. This could be attributed to the proximity of her home to the roadway emission source (the participant’s home is located within a few hundred meters from Interstate 2 freeway and two busy arterials).

## 5. Limitations of the Modeling Framework

Like any modeling analysis, the analysis presented in this paper has a number of assumptions and uncertainties. While quantifying these uncertainties is not within the scope of this study, an overview of the model assumptions and input data uncertainties associated with the analysis is provided in this section.

Firstly, the AERMOD model estimates concentration levels using a Gaussian steady-state formulation. This formulation assumes steady-state meteorological conditions to exist throughout the case study location and within each hour. In addition, Gaussian models are not capable of capturing the chemical transformation of pollutants and are applicable only to primary non-reactive pollutants. These models are thereby better suited for near-field situations where the conditions do not change significantly, such as in the case of traffic-related dispersion. Secondly, the average daily traffic volumes are converted using regional growth factors and traffic percentages. These regional factors are not site-specific and could introduce discrepancies in the emissions estimates and thereby exposure levels. Thirdly, concentration estimates for indoor and in-vehicle microenvironment are obtained from the modeled outdoor concentration combined with ratios from the literature. These ratios are not specific to the study area or the population sampled and could introduce discrepancies in the exposure estimates. Finally, the study did not include roadway emissions from the Mexico side of the US–Mexico border mainly because the impact from across the border is found to be beyond the near-road zone of influence in the study area. Vehicular emissions have a tendency to peak within a few hundred meters from the roadway edge and quickly drop to background levels. This near-zone of influence varies by pollutant and typically literature suggests this distance to be no more than 1640–3280 ft [75,76] for most pollutants. The closest distance between a participant location and the border highway is 1.69 m, which is beyond the near-zone of influence.

## 6. Conclusions

The integration of health and transportation considerations is a topic of growing importance to transportation researchers and health practitioners. Quantifying the contribution of traffic-related emission exposure in the overall population exposure is a key first step to developing targeted policy and interventions to address this issue. The current study presented a method for integrating the dynamics of modeled pollutant concentrations with the location information of participants tracked using GPS devices. The results exhibited a significant variation of emission exposure across time periods and spatial locations, which cannot be captured by simpler metrics such as traffic density and near-road distance. The study evaluated measures of static exposure based on residential location. The results exhibited the variation of traffic-related air pollution exposure across different microenvironments, which cannot be captured by simpler metrics such as traffic density and near-road distance. The study evaluated measures of static exposure based on residential location. Results showed a 9% increase in overall exposure predicted using the dynamic approach compared with the static approach. While prior studies indicated significantly larger differences between dynamic and static exposure measures, current results do not indicate a significant difference in accounting for the location information of participants in exposure assessment. However, these results must be interpreted in the specific context of this study, as the participants being in their later stages of gestation tended to increase the time spent at home.

The current study contributes to the literature on exposure assessment methods in important ways. Firstly, the dynamic exposure method developed based on GPS data and air-dispersion models offers several advantages over traditional exposure assessment methods. The method overcomes restrictions of ambient and personal monitoring in terms of the sample size, higher cost, equipment failure, pollutant type and averaging time measured. Secondly, the study explored the utility of emerging data collection technologies for analyzing people’s activity patterns. Thirdly, the study has demonstrated the ability to capture the exposure levels in a vulnerable population group in a previously understudied and economically disparate region in South Texas. This is one of the early few studies that examined the activity patterns and emission exposure levels for a vulnerable population of pregnant women. In spite of increasing evidence linking traffic-related exposure and birth defects, there are limited studies examining maternal exposure levels. Recommendations for future research include (1) evaluating the modeled exposure levels with other sources of data (such as personal monitoring and satellite data) and (2) extending beyond exposure assessment in terms of associating the different levels of pollutant exposure with the severity of health problems and conducting a full health risk characterization.

## Figures and Tables

**Table 1:** Microenvironmental adjustment factors for PM_2.5._

**Studies on Buildings**	**I/O Ratio**
[64]	0.88
[65]	0.73
[66]	0.84
[67]	1.06
[68]	0.67
[69]	0.995
**Studies on Vehicles**	**I/O Ratio**
[64]	0.85
[66]	2
[70]	0.76
[7]	2.68

**Table 2:** Traffic-related PM_2.5_ concentration (µg/m^3^) in three microenvironments over the 50 measurement days.

Micro- Environment	Traffic-Related PM_2.5_ Mass-to-Time Ratio	Traffic-Related PM_2.5_ Daily Mean (µg/m^3^)	Traffic-Related PM_2.5_ Standard Deviation	Range (µg/m^3^)
Indoor	0.91	0.29	0.21	0.02–0.92
Outdoor	1.45	0.26	0.27	0.00–1.61
Driving	1.96	0.56	0.55	0.04–2.26
Total		0.32	0.22	0.02–1.04
